# Current methods for developing predictive energy equations in maintenance dialysis are imprecise

**DOI:** 10.1080/07853890.2022.2057581

**Published:** 2022-03-31

**Authors:** Alainn Bailey, Rebecca Brody, Joachim Sackey, J. Scott Parrott, Emily Peters, Laura Byham-Gray

**Affiliations:** a Department of Clinical and Preventive Nutrition Sciences, School of Health Professions, Rutgers University, Newark, NJ, USA; b Department of Interdisciplinary Studies, School of Health Professions, Rutgers University, Newark, NJ, USA

**Keywords:** Resting energy expenditure, chronic kidney disease, predictive equation, haemodialysis, dialysis

## Abstract

**Purpose:**

For individuals receiving maintenance dialysis, estimating accurate resting energy expenditure (REE) is essential for achieving energy balance, and preventing protein-energy wasting. Dialysis-specific, predictive energy equations (PEEs) offer a practical way to calculate REE. Three PEEs have been formulated *via* similar methods in different demographic samples; the Maintenance Haemodialysis Equation (MHDE REE), Vilar et al. Equation (Vilar REE) and the Fernandes et al. Equation (Cuppari REE). We compared them in a US cohort and assessed precision relative to measured REE (mREE) from indirect calorimetry. Because of expected imprecision at the extremes of the weight distribution, we also assessed the PEEs stratified by body mass index (BMI) subgroups.

**Methods:**

This analysis comprised of 113 individuals from the Rutgers Nutrition and Kidney Database. Estimated REE (eREE) was calculated for each PEE, and agreement with mREE was set at > 50% of values within the limits of ±10%. Reliability and accuracy were determined using intraclass correlation (ICC) and a Bland Altman plot, which analysed the percentage difference of eREE form mREE.

**Results:**

Participants were 58.4% male and 81.4% African American. Mean age was 55.8 ± 12.2 years, and the median BMI was 28.9 (IQR = 25.3 − 34.4) kg/m^2^. The MHDE REE achieved 58.4% of values within ±10% from mREE; Cuppari REE achieved 47.8% and Vilar REE achieved 46.0% agreement. Reliability was good for the MHDE REE (ICC = 0.826) and Cuppari REE (ICC = 0.801), and moderate for the Vilar REE (ICC = 0.642) (*p* < .001 for all). The equations performed poorly at the lowest and highest BMI categories.

**Conclusion:**

Dialysis-specific energy equations showed variable accuracy. When categorized by BMI, the equations performed poorly at the extremes, where individuals are most vulnerable. Innovation is needed to understand these variances and correct the imprecision in PEEs for clinical practice.

**KEY MESSAGES**
- Potentially impacting over millions of patients worldwide, our long-term goal is to understand energy expenditure (EE) across the spectrum of CKD (stages 1–5) in adults and children being treated with dialysis or transplantation, with the intent of providing tools for the health professional that will improve the delivery of quality care.
- Our research has identified and focussed on disease-specific factors which account for 60% of the variance in predicting EE in patients on MHD, but significant gaps remain.
- Thus, our central hypotheses are that (1) there are unique disease-specific determinants of EE and (2) prediction of EE for individuals diagnosed with CKD can be vastly improved with a model that combines these factors with more sophisticated approaches.

## Introduction

According to the United States Renal Data System, almost 750,000 people in the US are diagnosed with stage 5 chronic kidney disease (CKD) on dialysis, which was a 91% increase since January 2000 [1]. Renal replacement therapy is a lifesaving treatment for these individuals, with 63% receiving maintenance haemodialysis (MHD) [1,2]. Despite ongoing improvements in survival, only 57% of those receiving MHD in 2011 were still alive three years after their first treatment [1]. The reasons for such high mortality are multifactorial. The leading cause is cardiovascular disease [3] followed by complications of diabetes [4]. As CKD progresses, compounded proinflammatory conditions, low protein, and energy intake, and reduced physical activity contribute to poor nutritional status and muscle catabolism [5–10]. This process can lead to protein-energy wasting (PEW), a condition unique to renal disease, further contributing to adverse health sequelae [5–10].

Establishing a person’s accurate energy expenditure is essential in setting nutritional goals, assessing if energy balance is achieved, aiding in determining an appropriate dose of dialysis, and is particularly important for those individuals at risk of PEW [7–13]. Indirect calorimetry (IC), which measures the exchange of inspired and expired gas to infer levels of cellular metabolism, is considered the gold standard for establishing an individual’s energy requirements [14]. However, conducting IC in the clinical setting is difficult as it increases the patient burden, and is hence infrequently used [15]. Studies have established that population-based predictive energy equations (PEEs), such as the Harris-Benedict Equation (HBE) [16] or Mifflin St Jeor (MJE) [17] are largely inaccurate in estimating resting energy expenditure (REE) of individuals requiring MHD as they fail to account for the differences in clinical characteristics [18–20].

In recent years, four dialysis-specific PEEs [21–24] have been developed in patient cohorts receiving MHD [21–24]. The equations developed by Vilar et al (Vilar REE) [23] and Fernandes et al (Cuppari REE) [21] use standard demographic and clinical measurements (age, height, weight, sex) to build their predictive algorithms [21,23]. The Maintenance Haemodialysis Equation (MHDE-REE) [22,24], first formulated by Byham-Gray et al in 2014 (and further refined in 2018), includes disease and inflammatory factors such as serum creatinine (SCr), C-reactive protein (CRP), and haemoglobin A1c (A1C) [22,24]. All of these PEEs were developed and tested in geographically different populations (England, USA and Brazil) comprising widely diverse racial and ethnic groups, where the critical determinants of estimated REE (eREE) such as average age, height, and weight varied. The Vilar et al. cohort was predominantly Caucasian [23], The Fernandes et al. cohort was Brazilian Hispanic [21], and the Byham-Gray et al. cohort was ostensibly African American [22,24]. The equations perform well in the populations for which they were developed, but few validation studies have compared them in groups that diverge from the development sample [21,25–27]. In 2019, Fernandes et al. compared the MHDE REE (SCr version), the Vilar REE, and the Cuppari REE in the Brazilian cohort used to create the Cuppari REE [21]. This study showed that the domiciliary PEE performed best (82.4% accurate between 80-120% of mREE) [21]. The MHDE REE and Vilar REE performed with less precision [21].

This study further explores the performance of the new PEEs in different populations by comparing the MHDE REE, Cuppari REE, and Vilar REE in a cohort of US patients receiving MHD. We hypothesised that the equations perform best in the samples from which they are derived. Furthermore, previous studies have established that BMI status may play a role in the precision of equations for specific individuals [23,26]. Adiposity, and the consequent rise in BMI, blunts the anticipated increase in REE due to disproportional changes in FFM to FM [23]. While FFM is the most precise parameter to use in PEEs, it is difficult to obtain accurately within the clinical setting. As such, weight is commonly used in PEEs secondary to its accessibility, and thereby introduces a level of error within the predictive modelling. Therefore, we have also analysed the performance of each equation within categories of BMI to better understand for whom the equations may perform best and worst.

## Methods

### Participants

This study was a secondary analysis of previously collected data mined from the *Rutgers Nutrition & Kidney Database* (RNKD) (Institutional Review Board protocol: 2020001656; approved as of 11/18/2020). The RNKD includes data from four existing studies 22 undertaken between 2012 and 2018. The studies all took place in the Northeastern/Midwestern regions of the United States. Sampling for enrolment was conducted on a convenience basis. Inclusion criteria comprised of adult men and women ≥ 18 years with stage 5 CKD receiving MHD 3 times weekly for a minimum of 3 months. All participants for this study had the data necessary to calculate all PEEs and a value for measured REE (mREE), the criterion standard. Exclusion criteria included contemporary infection or non-healing wounds, surgery within 30 days of enrolment, cardiovascular events within 30 days of enrolment, non-prescription drug usage, or quotidian dietary supplementation (where either impact metabolism), pre-existing cancer, heart failure, or hepatic disease [22].

### Data collection

All of the parent studies implemented similar data collection protocols. Demographic data were collected from individuals and medical records, and clinical/anthropometric data were collected on a non-dialysis day. Body weight was measured in pounds or kilograms. Height was assessed, without shoes, using a stadiometer. IC was conducted using a metabolic cart (Cosmed Quark RMR^®^, Rome, Italy). Participants were asked to refrain from vigorous physical activity and fast for 12 h before IC. If a 12 h fast was not achievable, a minimum fast of 4 h was requested. Fasting was implemented to minimise the effects of fluid accumulation on both weight and body composition. IC took place in the morning, in a comfortable room. Patients were recumbent, still, and awake for a minimum of twenty minutes. A plastic canopy was placed over the participant’s head to capture inspired and expired gas. The measurement was conducted for at least twenty minutes, and an abbreviated 5 min was documented at steady-state, with a variation coefficient of <10% [28]. Data utilised in this study included mREE, age, race, ethnicity, sex, dialysis vintage, weight, height, BMI, and clinical measurements (CRP, albumin).

### Measurement of estimated resting energy expenditure

The variables necessary to generate values for each equation (MDHE-REE, Vilar REE, Cuppari REE) were age, sex, weight, height (Vilar only), and CRP (MHDE only). Where the original authors provided multiple equations, the best clinical equation documented by study investigators was chosen. REE values were then calculated according to the appropriate equation (Table 1).

**Table 1.** Variables and Equations required to calculate MDHE REE, Vilar REE and Cuppari REE for women and men.

Equation	Variables required	Female Equation	Male Equation
MHDE REE	Age, weight, CRP, sex	REE = 820.47−(5.19*Age) + (9.67*weight) + (2.71*CRP)	REE = 1027.8 − (5.19*Age) + (9.67*weight) + (2.71*CRP)
Vilar REE	Age, weight, height, sex	REE = − 2.497 * Age * Factor_ag_*_e_* + 0.011 * Height^2.023^ +83.537 * Weight0.6291 + 68.1711 * Factor_female_	REE = − 2.497 * Age * Factor_ag_*_e_* + 0.011 * Height^2.023^ +83.537 * Weight0.6291 + 68.1711 * Factor_male_
Cuppari REE	Age, weight, sex	REE = 957.02 – (8.08 * Age) + (11.07* body weight)	REE = 957.02 − (8.08 * Age) + (11.07* body weight) + 136.4

CRP: C-reactive protein; REE: resting energy expenditure.

### Graphical and statistical analyses

A power analysis was completed in the original study that developed the MHDE [22]. We were able to show that *n* = 60 was sufficient to generate the equation and *n* = 95 was sufficient to validate the equation. As this study utilised the same dataset, with complete variables for *N* = 113, no further sample size analysis was undertaken. Additionally, findings achieved statistical significance demonstrating that the sample size was adequate.

All statistical analyses were undertaken on Statistical Package for Social Sciences (SPSS, IBM Corp., version 27, Armonk, NY). Normality was established by visual inspection of histograms, box and whisker, and q-q plots. The values were expressed as mean and standard deviation (SD), median, 25th and 75th percentiles, and minimum and maximum values. Intraclass correlation coefficient (ICC) was used to assess the reliability of each equation to replicate mREE. ICC estimates were calculated using a single rater, absolute agreement, 2-way mixed-effects model [29]. An *alpha priori* level was set at 0.05.

A modified version of the Bland-Altman plot assessed the level of agreement between mREE and eREE [30]. Bland-Altman analysis determines the agreement between two methods of measurement by graphically examining the model residuals taken by the two comparison methods (*Y*-axis) and the mean of the two measures (*X*-axis; assumed to be the “true” measure when the true measure is unknown) . For this study, the relationship between eREE and mREE was examined. The plots were generated by comparing residual (eREE-mREE) expressed as a percentage of mREE ((eREE-mREE)/mREE {*Y*-axis}), which allowed measurement of variations in agreement along the total distribution of mREE values (*X*-axis). In contrast to the typical Bland-Altman approach, the limits of agreement were set at ±10% from zero difference from mREE, as established in the nutrition literature regarding validation of predictive energy equations [31] and applied by Byham-Gray et al. [22,24] and Morrow et al. [26] for individuals receiving MHD in particular [22,24,26,31]. Modified Bland-Altman plots were generated for each equation using the total sample, then the percentage of values falling within ±10% from zero difference was calculated. If 50% or more of the measurements fell within the ±10% band of confidence, then eREE was deemed to show overall agreement with mREE. Thereafter, the analysis was repeated for each PEE with the sample stratified by BMI subgroups. Individuals with a BMI less than 24.9 kg/m^2^, 25–29.9 kg/m^2^, or ≥30 kg/m^2^ were categorised as underweight/normal weight, overweight, or obese.

## Results

This study sample (*N* = 113) was 58.4% male, 76.1% non-Hispanic, and 81.4% African American (Table 2). The individuals’ ages ranged between 21.5 and 80.7 years, with a mean age of 55.8 ± 12.2 years. The median BMI of individuals was 28.9 (IQR = 25.3–34.4) kg/m^2^, with 23.9% categorised as underweight or normal weight, 36.3% categorised as overweight, and 39.8% categorised as obese (Tables 2 and 3). Median CRP was within normal limits, although 68.1% (*n* = 77) of participants reported CRP levels above 2.0 mg/L, indicating low-grade systemic inflammation and a higher risk for chronic disease [32].

**Table 2.** Frequency of clinical and demographic characteristics of individuals in the Rutgers Nutrition and Kidney Database (N = 113).

Variable	*n*	%
Sex		
Male	66	58.4
Female	47	41.6
Ethnicity		
Non-Hispanic	86	76.1
Hispanic	10	8.8
Unknown	17	15.0
Race		
African American	92	81.4
White	21	18.6
BMI		
Underweight/Normal weight (< 24.9 kg/m^2^)	27	23.9
Overweight (25–29.9 kg/m^2^)	41	36.3
Obese (≥ 30 kg/m^2^)	45	39.8

BMI: body mass index; kg/m^2^: kilograms per metre squared.

**Table 3.** Demographic and clinical characteristics among individuals in the Rutgers Nutrition and Kidney Database (N = 113).

Variable	*n*	Mean ± SD	Median (25–75th percentiles)	Minimum–maximum
Age (years)		55.8 ± 12.2	55.8 (48.4 − 63.7)	21.5 − 80.7
Weight (kg)		86.5 ± 21.0	84.4 (71.8 − 98.8)	47.1 − 150.8
Height (cm)		169.4 ± 10.1	170.9 (162.0 − 177.0)	143.9 − 193.6
BMI (kg/m^2^)		30.1 ± 6.8	28.9 (25.3 − 34.4)	18.7 − 50.8
CRP (mg/L)		10.5 ± 14.9	5.4 (1.3 − 12.0)	0.1 − 93.0
Albumin (g/dL)		4.2 ± 0.4	4.2 (3.9 − 4.5)	3.1 − 5.4
Dialysis Vintage (months)		61.1 ± 69.6	42.0 (21.0 − 84.0)	3.5 − 411.0
mREE (kcal)		1521.8 ± 334.4	1448.6 (1296.7 − 1684.3)	880.6 − 2448.1
Male	66	1660.8 ± 331.2	1588.8 (1427.3 − 1859.7)	880.6 − 2448.1
Female	47	1326.5 ± 225.1	1327.7 (1127.0 − 1478.7)	995.0 − 1873.6
<65 years	87	1581.8 ± 335.8	1509.0 (1337.1 − 1829.7)	1028.2 − 2448.1
≥65 years	26	1320.9 ± 242.2	1296.6 (1091.6 − 1543.1)	880.6 − 1688.6
MHDE-CRP REE (kcal)		1516.5 ± 284.4	1485.8 (1334.4 − 1701.8)	882.4 − 2340.8
Male	66	1652.9 ± 244.8	1606.3 (1484.4 − 1756.8)	1241.6 − 2340.9
Female	47	1325.0 ± 219.2	1316.0 (1192.3 − 1419.5)	882.4 − 1969.0
<65 years	87	1575.5 ± 276.6	1508.9 (1394.0 − 1733.7)	1057.4 − 2340.8
≥65 years	26	1319.1 ± 215.5	1336.4 (1146.6 − 1490.8)	882.4 − 1727.4
Vilar REE (kcal)		1728.0 ± 271.5	1692.5 (1558.1 − 1922.0)	1038.4 − 2417.4
Male	66	1827.6 ± 238.9	1801.4 (1639.7 − 2011.2)	1326.4 − 2417.4
Female	47	1588.2 ± 254.2	1502.8 (1407.2 − 1703.4)	1038.4 − 2246.5
<65 years	87	1805.1 ± 242.1	1746.2 (1627.8 − 2000.9)	1308.3 − 2417.4
≥65 years	26	1407.1 ± 196.9	1495.7 (1337.3 − 1800.2)	1038.4 − 1772.8
Cuppari REE (kcal)		1543.1 ± 300.0	1510.0 (1351.0 − 1712.1)	863.8 − 2522.3
Male	66	1653.0 ± 279.5	1594.2 (1446.8 − 1793.5)	1149.0 − 2522.3
Female	47	1388.6 ± 258.8	1368.2 (1217.9 − 1519.1)	863.8 − 2124.9
<65 years	87	1617.1 ± 286.3	1551.5 (1429.9 − 1782.8)	1010.7 − 2522.3
≥65 years	26	1295.5 ± 195.8	1304.1 (1148.6 − 1455.4)	863.8 − 1587.9

BMI: body mass index; cm: centimetres; CRP: C-reactive protein; g/dL: grams per decilitre; IQR: interquartile range; kcal: kilocalories; kg: kilograms; kg/m^2^: kilograms per metre squared; Max: maximum; Min: minimum; MHDE-CRP REE: maintenance haemodialysis C-reactive protein equation for resting energy expenditure; mREE: measured resting energy expenditure; REE: resting energy expenditure; SD: standard deviation.

### Measured and predicted energy requirements

The median mREE was 1448.6 kcal/day and ranged from 880.6 to 2448.1 kcal (Table 3). Median mREE for women (1327.7 kcal/day) and those 65 years and above (1336.4 kcal/day) was lower than for men (1588.8 kcal/day) and for younger individuals (1509.0 kcal/day). The MHDE REE had the lowest average prediction of energy requirements, with a median eREE of 1485.8 kcal/day.

### Levels of agreement

**Figure 1.**
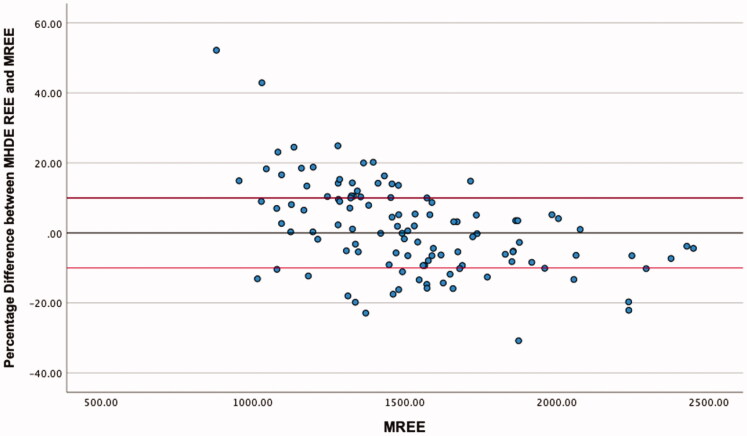
Modified Bland Altman Plot of the percentage difference between The MHDE REE and mREE. The black line represents zero difference from mREE. The upper red line represents 10% difference from mREE. The lower red line represents −10% difference from mREE.

**Figure 2.**
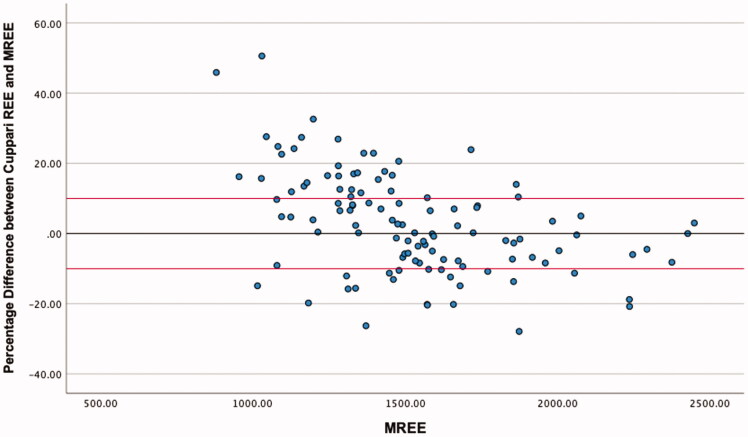
Modified Bland Altman Plot of the percentage difference between The Cuppari REE and mREE. The black line represents zero difference from mREE. The upper red line represents 10% difference from mREE. The lower red line represents −10% difference from mREE.

**Figure 3.**
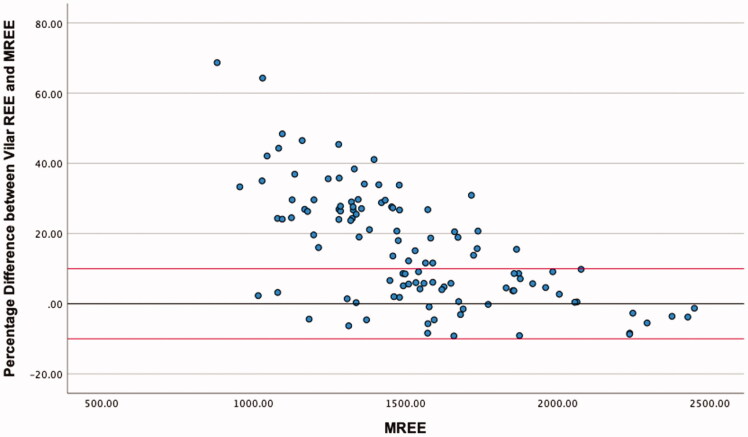
Modified Bland Altman Plot of the Percentage Difference between The VIlar REE and mREE. The black line represents zero difference from mREE. The upper red line represents 10% difference from mREE. The lower red line represents −10% difference from mREE.

The highest level of agreement was between mREE and the MHDE REE, with 58.4% of values falling within the limits of acceptability (Table 4). Neither of the other equations met the 50% threshold for acceptable values. ICC estimates indicated good reliability for the MHDE REE and the Cuppari REE, and moderate reliability for the Vilar REE. Visual inspection of the modified Bland-Altman plots indicates that eREE values ±10% from mREE are evenly distributed for the MHDE REE (Figure 1) and Cuppari REE (Figure 2) and that the Vilar REE tends to overestimate in greater than 50% of individuals (Figure 3).

**Table 4.** Levels of agreement in resting energy expenditure as derived by indirect calorimetry, compared to three predictive energy equations for individuals receiving maintenance haemodialysis (N = 113).

Equation	Withi*n* ± 10% of mREE *n* (%)	<10% of mREE *n* (%)	> 10% of mREE *n* (%)	Intraclass Correlation R	*p* Value
MHDE REE	66 (58.4)	25 (22.1)	22 (19.5)	0.826	<.001
Vilar REE	52 (46.0)	0 (0.0)	61 (53.0)	0.642	<.001
Cuppari REE	54 (47.8)	22 (19.5)	37 (32.7)	0.801	<.001

MHDE REE: maintenance haemodialysis C-reactive protein equation for resting energy expenditure; mREE: measured resting energy expenditure; REE: resting energy expenditure.

### Variability of agreement in different categories of BMI

**Figure 4.**
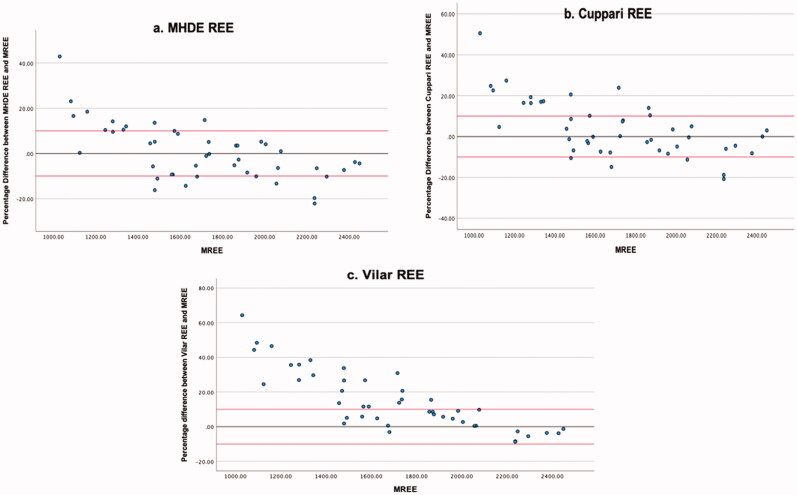
(a–c) Obese. Percentage Difference Between Three Different MHD PEE’s and mREE in people receiving MHD Categorised as *Obese*. The black lines represent zero difference from mREE. The upper red lines represent 10% difference from mREE. The lower red lines represent −10% difference from mREE.

For participants with a BMI categorised as obese, the MHDE REE and Cuppari REE demonstrated equal levels of accuracy (57.8% within limits) (Table 5). The Vilar REE predicted 51.1% of estimates within acceptable limits, and those estimates outside of the acceptable limit of ±10% tended to overestimate REE (22.2%, 31.1%, and 48.9%, respectively) (Figure 4(a–c)).

**Table 5.** Levels of agreement in resting energy expenditure as derived by indirect calorimetry, compared to three predictive energy equations for individuals with a *BMI over 30 kg/m^2^* receiving maintenance haemodialysis (*n* = 45).

Equation	Within ±10% of mREE *n* (%)	<10% of mREE *n* (%)	>10% of mREE *n* (%)	Intraclass correlation *R*	*p* Value
MHDE REE	26 (57.8)	9 (20.0)	10 (22.2)	0.853	<.001
Vilar REE	23 (51.1)	0 (0.0)	22 (48.9)	0.669	<.001
Cuppari REE	26 (57.8)	5 (11.1)	14 (31.1)	0.826	<.001

MHDE REE: maintenance haemodialysis C-reactive protein equation for resting energy expenditure; mREE: measured resting energy expenditure; REE: resting energy expenditure.

**Figure 5.**
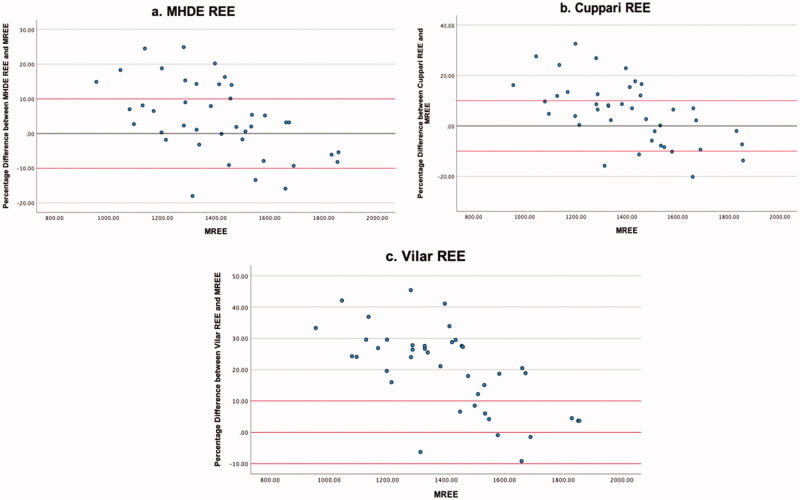
(a–c) Overweight. Percentage Difference Between Three Different MHD PEE’s and mREE in people receiving MHD Categorised as *Overweight*. The black lines represent zero difference from mREE. The upper red lines represent 10% difference from mREE. The lower red lines represent −10% difference from mREE.

For participants with a BMI categorised as overweight, accuracy was greater for the MHDE REE (63.4% within limits) and the Cuppari REE (56.1% within limits) than when the entire study sample was analysed (Table 6), and accuracy was reduced for the Vilar REE (26.8% within limits). Again all of the PEEs tended to overestimate eREE where values did not fall within acceptable limits (29.3%, 31.7%, and 73.2%, respectively) (Figure 5(a–c) and Table 6).

**Table 6.** Levels of agreement in resting energy expenditure as derived by indirect calorimetry, compared to three predictive energy equations for individuals with a *BMI between 25 and 30 kg/m^2^* receiving maintenance haemodialysis (*n* = 41).

Equation	Within ± 10% of mREE *n* (%)	<10% of mREE *n* (%)	>10% of mREE *n* (%)	Intraclass correlation *R*	*p* Value
MHDE-CRP	26 (63.4)	3 (7.3)	12 (29.3)	0.771	<.001
Vilar REE	11 (26.8)	0 (0.0)	30 (73.2)	0.391	<.001
Cuppari REE	23 (56.1)	5 (12.2)	13 (31.7)	0.635	<.001

MHDE-CRP REE: maintenance haemodialysis C-reactive protein equation for resting energy expenditure; mREE: measured resting energy expenditure; REE: resting energy expenditure.

**Figure 6.**
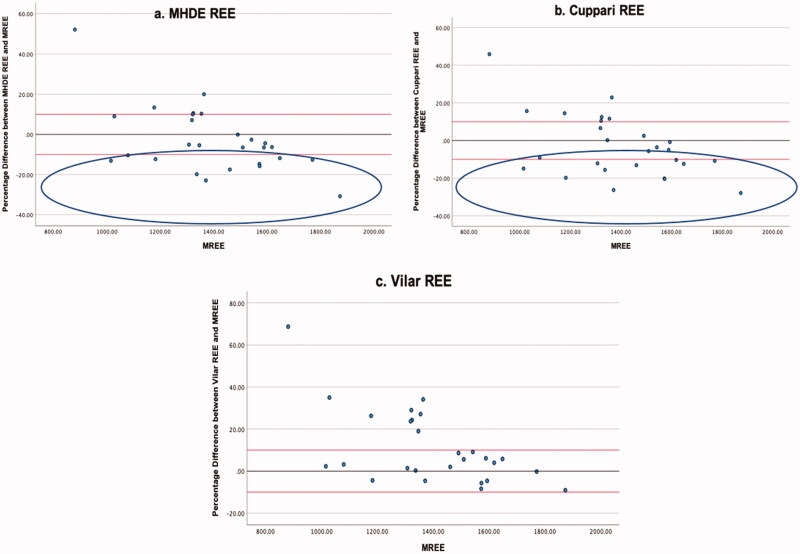
(a–c) Underweight. Percentage Difference Between Cuppari REE and mREE in people receiving MHD Categorised as *Normal Weight or Underweight*. The black lines represent zero difference from mREE. The upper red lines represent 10% difference from mREE. The lower red lines represent −10% difference from mREE. The blue oval highlights those individuals for whom REE is underestimated by more than 10% from mREE.

For individuals with a BMI defined as normal or underweight, accuracy did not meet the 50% threshold for agreement for the MHDE REE (40.7% within limits) and the Cuppari REE (29.6% within limits) (Table 7). Additionally, both the MHDE REE and the Cuppari REE tended to underestimate eREE relative to mREE (40.7% and 44.4%, respectively) (Figure 6(a–c) and Table 7). The Vilar REE performed best with 66.7% of estimates within the limits of acceptability in normal and underweight individuals. In this category, none of the equations demonstrated good reliability as measured by ICC.

**Table 7.** Levels of agreement in resting energy expenditure as derived by indirect calorimetry, compared to three predictive energy equations for individuals with a *BMI below 25 kg/m^2^* receiving maintenance haemodialysis (*n* = 27).

Equation	Withi*n* ± 10% of mREE *n* (%)	<10% of mREE *n* (%)	> 10% of mREE *n* (%)	Intraclass correlation *R*	*p* Value
MHDE-CRP	11 (40.7)	11 (40.7)	5 (18.5)	0.522	.002
Vilar REE	18 (66.7)	0 (0.0)	9 (33.3)	0.517	<.001
Cuppari REE	8 (29.6)	12 (44.4)	7 (25.9)	0.522	.002

MHDE-CRP REE: maintenance haemodialysis C-reactive protein equation for resting energy expenditure; mREE: measured resting energy expenditure; REE: resting energy expenditure.

## Discussion

We compared mREE derived from IC to the outputs of three PEEs: the MHDE REE, the Vilar REE, and the Cuppari REE. We found that only the MHDE produced eREE values within the limits of agreement (>50% of values ±10% of zero difference) in this sample of individuals.

We also analysed the levels of agreement between mREE and the PEEs when the dataset was categorised by BMI status to explore the performance of the models in different weight categories. This analysis showed that none of the equations perform well in every category. Of particular concern, the Cuppari REE and MHDE REE tended to underestimate REE for the normal weight and underweight category, including the individuals most at risk for protein-energy wasting.

Previous studies (Vilar et al., Byham Gray et al., Fernandes et al.) [21,23–25] have demonstrated that MHD-specific equations estimate REE accurately for >50% of the individuals within their own specific cohorts, although limits of agreement vary, with subsequent impacts on the levels of precision found. This study applied consistent limits of agreement (±10%) to all the PEEs and found that only the MHDE estimated REE accurately for greater than 50% of individuals. We utilised the same cohort as that used to generate the MHDE, with the likely outcome that specific traits of the domicile cohort account for the superior performance of the MHDE in our study.

Hung et al. [27] and Oliviera et al. [25] validated the Vilar REE in an English cohort using bioimpedance analysis (BIA) and found that the Vilar REE predicted higher energy expenditure than PEEs for the general population and correlated more closely with REE predicted *via* BIA using the Cunningham and Katch-Mc-Ardle equations [25,27]. Although BIA provides a more objective estimate of REE, it is not considered the criterion measure, as is IC. Of note, the Vilar REE performed well in these investigations using English study groups similar to the original Vilar cohort [25,27]. Morrow et al. [26] assessed the MHDE REE (SCr version) and the Vilar REE in a small US cohort using a handheld IC device (which also is not a criterion measure of REE) [26]. Morrow and colleagues found that the MHDE REE performed well (52.5% agreement), but the Vilar REE did not [26]. In the Morrow et al. study, the Texas-based cohort shared similar characteristics to our study group, including key variables such as age (56.7 years), racial mix (85% African American), and mean BMI (29.7) [26]. Fernandes et al. [21] demonstrated that their own equation (the Cuppari REE) provided greater than 80% accuracy within a Brazilian cohort with similar characteristics to their original study group [21]. The authors also assessed the MHDE REE and Vilar REE performance in the Brazilian cohort and found low to moderate accuracy [21]. At face value, the level of precision for the Cuppari REE appears almost twice as high in the Brazilian study, however, the investigators used broader bands of agreement (±20%) versus our own (±10%) [21]. Our findings are consistent with the existing literature, demonstrating that the three MHD PEEs perform best in samples with similar characteristics to the cohorts in which the equations were constructed. Moreover, using linear methods, the current equations do not perform well in groups of people with different characteristics, and a more innovative statistical approach should be considered.

### Exploring the impact of common variables on predictive performance

Looking at the equations themselves, individual variables may explain divergent performance; e.g. the application of age and sex. As reported by all four studies that constructed PEEs [21–24], age was a strong negative predictor of REE, and was therefore used by each author in equation building [21–24]. However, study groups differed in average age, which resulted in differing correlation coefficients between the studies (*r* ranged from −0.33 [21] to −0.46 [24]), and resulted in different age coefficients within each model [21–24]. The MHDE REE and Cuppari REE, with younger cohorts, applied a linear age adjustment for all participants. With an older cohort, the Vilar REE applied age as a factor only to those older than 65 years. When the Vilar equation was then used to calculate eREE in our younger study population, the average age adjustment to eREE was considerably lower. Divergent calorie adjustments were also allocated between men and women. In every equation, the assumed metabolic difference was applied as a single value for the standard woman and man. Thus, women using the Vilar equation expend only 68.1 kcal less than men, compared to 207.3 kcal assumed by the MHDE. More sophisticated statistical methods are required to explore the interactions of such variables before they can be generally applied to all individuals.

### Assessing the importance of clinical variables: CRP

In attempting to explain variance in mREE better, the MHDE REE includes increased metabolic expenditure for clinical factors such as inflammation (CRP), diabetes status (A1c), and muscle catabolism (SCr) [22]. Our study used the MHDE CRP version, which may have increased precision in this study group where inflammation was largely elevated. Other studies predicting REE in patients receiving MHD have found conflicting results for the impact of inflammation. For example, Byham-Gray et al. [22,24] and Fernandes et al. [21] identified CRP to be positively correlated with REE [21,22,24]. Vilar et al. [23] and Hung et al. [27] did not identify a correlation, despite having similar levels of CRP to our study group [23,27]. This points to how other differences in study group characteristics interact with the relative frequency of underlying comorbidities and disease severity and how this may be captured in future equations. More fundamentally, it requires markers such as CRP to be routinely measured in the clinic.

### Precision and BMI status

This is the first known study to analyse the output of all PEEs by BMI category, which provided some distinguishing insights. Morrow et al. [26] observed that overestimation of REE was more likely at the lower end of the distribution of REE values, and underestimation was more likely at the higher end [24]. Our study confirmed Morrow’s findings in relation to absolute energy expenditure (Figures 1 and 2) but not when the data were stratified by BMI. The MHDE REE and the Cuppari REE performed adequately for individuals who were overweight or obese but performed poorly for individuals who were underweight or normal weight. Both equations tended to underestimate the REE of patients in the lower BMI category and overestimate the REE of patients who were overweight or obese. BMI differences provide a further example of the limitations in the generalisability of cohort characteristics and equation methodologies. Weight is used as the predominant predictor of REE, as FFM is rarely available in the clinic [21–24]. Height is also an independent variable, despite much collinearity [21–23]. Therefore, the intersection of weight and height in a particular cohort will affect the parameters and performance of the models. Equations generated from a sample of people who are predominantly obese will perform best when predicting the REE of other people with obesity and vice versa. The MHDE REE, generated from a sample with a BMI around 30 kg/m^2,^ performed well for individuals with a similar BMI but poorly for individuals who were underweight or normal weight. This group includes vulnerable people, such as those with protein-energy wasting or other catabolic complications, which has important implications for the clinical decision-making process.

### Other methodological differences

Other methodological differences may account for predictive variation in the three PEEs. The individuals used to develop Fernandes et al.’s equation completed a 12-h overnight fast before IC measurement was made [21]. In the Byham-Gray et al. cohort, a 12-h fasting was requested, but, if not feasible, a 4-h fast was accepted [24]. In the Vilar et al. cohort, fasting was limited to 2 h [23]. Additionally, equations were formulated within different mixtures of patients receiving MHD and/or PD [21,23,24]. These differences may have contributed to altered metabolic activity and mREE.

Fernandes et al. used FFM in one of their equations to assess if this variable was better than body weight; however, no improvement in precision was found [21]. Obtaining accurate weight, muscle mass, and fluid status remain a constant challenge in this population.

### Limitations of the study

This study used a non-random, convenience sample recruited in a narrow geographic area. Participants were mostly male, African American, and younger than the national average of individuals receiving MHD. The original study imposed strict criteria, which excluded sicker individuals and the sample size was small. These factors limit the generalisability of the results. IC and anthropometric measurements were conducted on a non-dialysis day. This suggests that weight would vary from post-dialysis weight, depending on the individual’s fluid intake and residual ability to void, and may impact BMI status. Finally, although the level of 50% agreement within 10% of zero difference has been established as the criterion methodology for comparing eREE to mREE , it may be argued that a disease-specific PEE should perform with greater precision within the particular population for which it is designed.

### Implications for practice and research

The application of PEEs in the clinical setting allows for rapid calculation of energy requirements to promote optimal nutritional balance and care. This study indicates that clinicians should selectively and cautiously apply PEEs, especially where patients exhibit unique characteristics. If the patient is underweight, unintentionally losing weight, has a diagnosis of PEW or requires precise individualised care, such as nutrition support, it may be more appropriate to assess the patient using IC.

The authors of the research reviewed in this study have made significant improvements in predicting energy expenditure in patients receiving MHD, but challenges and gaps remain. For example, the RNKD database was limited to ambulatory patients who were African American, younger, and healthier. The recruitment of larger cohorts that better represent national and international patients’ demographic and clinical diversity is required before equations can be fully validated and improved. Notwithstanding, advances in technology and computational capabilities now allow researchers to better unravel the complex relationships of key anthropometric and clinical variables. The widespread availability of technology means that more complex algorithms can be applied to clinical practice *via* smartphones or tablets. The next step from this study is to quantify the interactions between variables that contribute to the poor performance of PEEs in vulnerable subgroups and tailor energy predictions to the individual patient.

### Conclusion

Establishing accurate energy expenditure in patients receiving MHD is essential to setting nutritional goals, assessing energy balance, and supporting health status and dialysis tolerance. This is especially important for those at high risk of catabolism and protein-energy wasting. Disease-specific PEEs, using simple variables and linear regression techniques, predict REE with fair accuracy when applied to people with similar characteristics as the development cohort. They do not perform well when applied to populations with differing characteristics or individuals with outlying characteristics that indicate an increased health risk. With technological advances becoming increasingly available and inexpensive, these findings highlight the need for greater individualisation in the estimation of REE, either *via* the development of more sophisticated equations or the direct application of IC for the most vulnerable patients.

## Data Availability

The data that support the findings of this study are available on request from the corresponding author, Laura Byham-Gray, and require a data sharing agreement. The data are not publicly available due to restrictions, i.e. the data may contain information that could compromise the privacy of research participants.
